# Evaluation of diethyl 4-(5-bromo-1H-indol-3-yl)-2,6-dimethyl-1,4-dihydropyridine-3,5-dicarboxylate: synthesis, anti-corrosion potential, and biomedical applications

**DOI:** 10.1186/s13065-024-01123-4

**Published:** 2024-05-10

**Authors:** F. M. Mashood Ahamed, M. Syed Ali Padusha, A. Mushira Banu, Swastika Maitra, Hanan M. Alharbi, Vinoth Kumarasamy, Daniel E. Uti, Popat Mohite, Athanasios Alexiou, Iftikhar Ali

**Affiliations:** 1 PG and Research Department of Chemistry, Jamal Mohamed College (Autonomous), Affiliated to Bharathidasan University, Tiruchirappalli, Khajanagar 620020 India; 2 Center for Global Health Research, Saveetha Medical College and Hospital, Saveetha Institute of Medical and Technical Sciences, Chennai, India; 3 Department of Pharmaceutics, College of Pharmacy, Umm Al-Qura University, 21955 Makkah, Saudi Arabia; 4 Department of Parasitology and Medical Entomology, Faculty of Medicine, Universiti Kebangsaan Malaysia, Jalan Yaacob Latif, Cheras, 56000 Kuala Lumpur, Malaysia; 5 Department of Biochemistry, Faculty of Basic Medical Sciences, College of Medicine, Federal University of Health Sciences, Otukpo, Benue Nigeria; 6 AETs St, John Institute of Pharmacy and Research, Palghar, 401 404 India; 7 Department of Science and Engineering, Novel Global Community Educational Foundation, Hebersham, NSW 2770 Australia; 8 AFNP, 1030 Wien, Austria; 9 Department of Genetics and Development, Columbia University Irving Medical Center, New York, NY 10032 USA

**Keywords:** Dihydropyridine, Corrosion, Mild steel, Antimicrobial, Antioxidant, Free radical

## Abstract

The pursuit of advanced multifunctional compounds has gained significant momentum in recent scientific endeavours. This study is dedicated to elucidating the synthesis, rigorous characterization, and multifaceted applications—encompassing anti-corrosion, antimicrobial, and antioxidant properties—of Diethyl 4-(5-bromo-1H-indol-3-yl)-2,6-dimethyl-1,4-dihydropyridine-3,5-dicarboxylate. The 1,4-dihydropyridine derivative was meticulously synthesized through a strategic reaction of ethyl acetoacetate, ammonium acetate, and 5-bromoindole-3-carboxaldehydein the ethanol medium at 60  C. Subsequent spectral validations were conducted using sophisticated techniques, namely FTIR, NMR, and Mass spectrometry, resulting in data that perfectly resonated with the hypothesized chemical structure of the compound. Its anti-corrosive potential was assessed on mild steel subjected to an aggressive acidic environment, employing comprehensive methodologies like gravimetric analysis, Tafel polarization, and EIS. Concurrently, its antimicrobial prowess was ascertained against a spectrum of bacterial and fungal pathogens viz., *Staphylococcus aureus, Bacillus subtilis, Escherichia coli, Pseudomonas, Candida albicans*and*Aspergillusniger*, leveraging the disc diffusion method and using Gentamicin as a reference standard.The empirical results illustrated a substantial decrement in corrosion rates with ascending concentrations of the organic compound, achieving an apex of anti-corrosive efficacy at 81.89% for a concentration of 2 × 10^3^ M. Furthermore, the compound outperformed Gentamicin in antimicrobial screenings, manifesting superior efficacy against all tested pathogens. The antioxidant potential, quantified using the DPPH free radical scavenging assay against ascorbic acid as a benchmark, was found to have an IC_50_ value of 113.964 ± 0.076 µg/ml.This comprehensive investigation accentuates the paramount potential of the synthesized dihydropyridine derivative in diverse domains—from industrial applications as a corrosion inhibitor to therapeutic avenues given its pronounced antimicrobial and antioxidant capabilities. The compelling results obtained pave the way for expansive research and development initiatives cantered around this multifaceted compound.

**Graphical Abstract:**

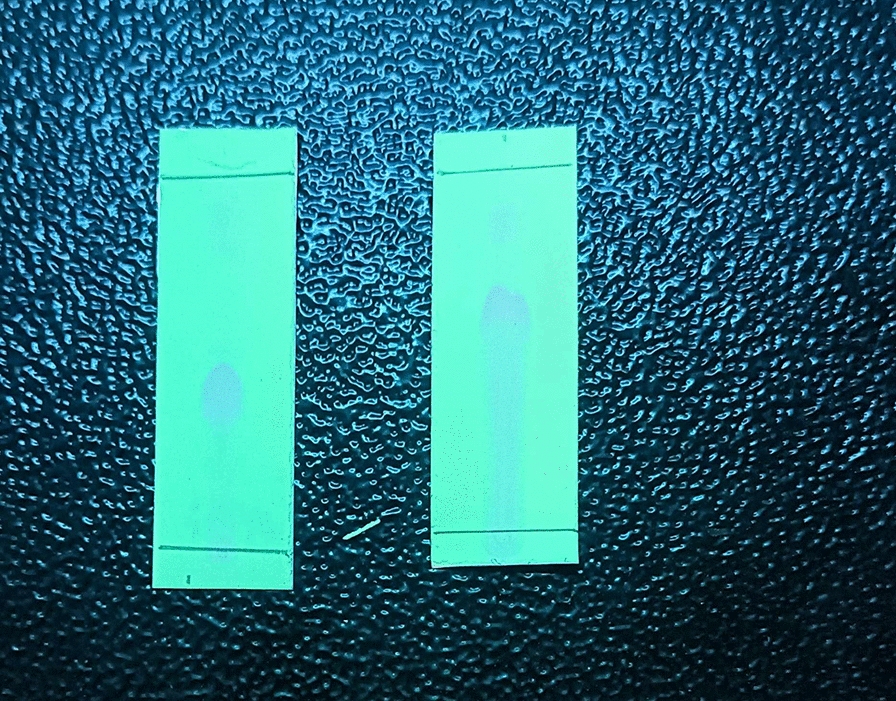

## Introduction:

Every living cell is a dynamic entity, teeming with molecular reactions, many of which produce reactive oxygen species (ROS) and free radicals as by-products. These by-products, while a natural result of cellular metabolism, become problematic when their production outpaces the body's capability to neutralize or remove them. The deleterious effects of these unchecked free radicals involve structural damage to cellular components, degradation of tissue matrices, and harmful mutations within DNA sequences [1]. Such oxidative disruptions precipitate a host of downstream complications, most notably inflammation, which serves as a common underpinning in numerous chronic diseases such as diabetes, cardiovascular ailments, and cancers. Antioxidants emerge as our primary defence mechanism in this context, scavenging these free radicals, mitigating oxidative stress, and restoring cellular balance.The vast pharmacopeia of cardiovascular therapeutics introduced us to Dihydropyridines (DHPs), a class of compounds renowned for their calcium antagonist properties. Their flagship representation, Lacidipine, among others, has been pivotal in hypertensive patient management [2]. However, recent forays into the deeper pharmacodynamics of DHPs have unveiled an intriguing secondary profile: their capability to act as antioxidants [3, 4]. This dual activity postulates DHPs as not only pivotal cardiovascular agents but also as potential therapeutic agents against diseases borne from oxidative stress.

In parallel to the biomedical world's battle against oxidative stress, the industrial sector faces a perennial challenge against metal corrosion. Ensuring the structural integrity of machinery, pipelines, and infrastructure hinges on effective corrosion management. At the molecular forefront of this battle are compounds featuring conjugated systems, π-electron configurations, and hetero atoms like Nitrogen (N), Oxygen (O), and Sulfur (S). Their efficacy as corrosion inhibitors is rooted in their ability to form protective interactions with metal surfaces, especially through electrostatic forces with Fe2 + ions [5–8]. An expanding repertoire of synthesized heterocyclic compounds have proven their mettle in varied corrosive environments, either through direct electron donation or via establishing electrostatic equilibriums at the metal-solution interface [5, 9–16].

Motivated by the critical roles of antioxidants in biomedicine and corrosion inhibitors in industry, this research pivots on a detailed investigation of Diethyl 4-(5-bromo-1H-indol-3-yl)-2,6-dimethyl-1,4-dihydropyridine-3,5-dicarboxylate. By synthesizing and characterizing this compound, and subsequently evaluating its anticorrosion, antimicrobial, and antioxidant attributes, we aim to carve a niche for its potential applications, potentially bridging biomedical therapeutics and industrial applications.

## Materials and methods

High purity chemicals were integral to the synthesis and ensuing applications to ensure that no confounding variables were introduced during the experiments. We employed ammonium acetate, ethyl benzoyl acetate, and 5-bromoindole-3-carbaldehyde sourced from a reputable supplier, Alfa Aesar, known for its high-grade chemicals.The solvents used for dilution were of spectroscopic grade, ensuring that they did not contribute any significant peaks or noise during spectral analysis. Prior to usage, these solvents underwent a decontamination process following established protocols to eliminate any potential contaminants.

### For spectral studies

- **FT-IR Spectra:** The KBr disc method, a popular choice for sample preparation due to its ability to produce high-quality spectra, was employed. Analysis was conducted on a state-of-the-art Shimadzu FT-IR spectrometer, scanning a range between 400 and 4000 cm^ −1^ to capture a comprehensive profile of the compound.
- ^**1**^**H-NMR Spectra:** Utilizing a Bruker 400 MHz spectrophotometer, which is recognized for its high-resolution outputs, we recorded the spectra in d6-DMSO solvent. Tetramethylsilane (TMS) was chosen as the internal standard because of its inertness and distinct peak.
- **Mass Spectra:** Electron Ionization (EI) method was chosen for its efficiency in ionizing molecules. High-Resolution Mass Spectroscopy (HRMS) measurements were made on a JEOL GC mate II, renowned for its accuracy and precision.

### Synthesis of Diethyl 4-(5-bromo-1H-indol-3-yl)-2,6-dimethyl-1,4-dihydropyridine-3,5-dicarboxylate

The synthesis began by dissolving ammonium acetate (7.7 g, 0.1 mol) in ethanol (20 ml). The choice of ethanol as a solvent provides a conducive environment for the reaction due to its polar nature and ability to dissolve a wide range of organic compounds. Subsequently, ethyl acetoacetate (12.8 mL, 0.1 mol) was introduced, providing the necessary carbonyl functionality to the reaction mixture. This was followed by the addition of 5-bromoindole-3-carboxaldehyde (22.4 g, 0.1 mol), which serves as the key aromatic component in the synthesis.

**Fig. 1:**
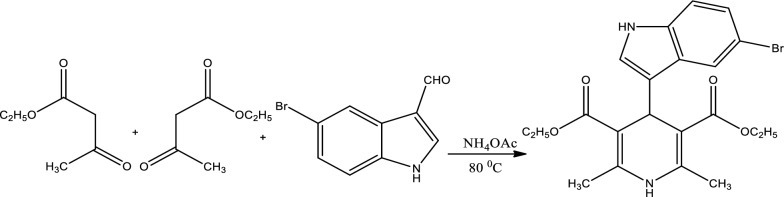
Synthesis of Diethyl 4-(5-bromo-1H-indol-3-yl)-2,6-dimethyl-1,4-dihydropyridine-3,5-dicarboxylate

The mixture was then refluxed for 6 h. Refluxing ensures that the reaction proceeds to completion by maintaining a constant temperature and preventing the loss of volatile reactants or products. Upon completion, the reaction mixture was quickly cooled by pouring it onto crushed ice, a method to facilitate the precipitation of the desired product. The resultant solid was then isolated using a filtration technique, ensuring the removal of any unreacted compounds or by-products. To further enhance the purity, the compound was recrystallized using absolute ethanol, which can remove any adhered impurities and also aids in obtaining a crystalline product.The structural and physical properties of the synthesized compound were then recorded and are available in Fig. 1 and Table 1 respectively.

**Table 1:** Conventional gravimetric data

Inhibitor Concentration, M	303 K	
	C.R (g cm^−2^ h^−1^)	I.E (%)
Blank	0.9271	-
0.00004	0.8614	07.09
0.00008	0.7081	23.62
0.00012	0.5840	37.00
0.00016	0.3723	59.84
0.00020	0.1679	81.89

### Spectral characterization of Diethyl 4-(5-bromo-1H-indol-3-yl)-2,6-dimethyl-1,4-dihydropyridine-3,5-dicarboxylate (DBIDDD)

#### ^1^H-NMR analysis

**Fig. 2:**
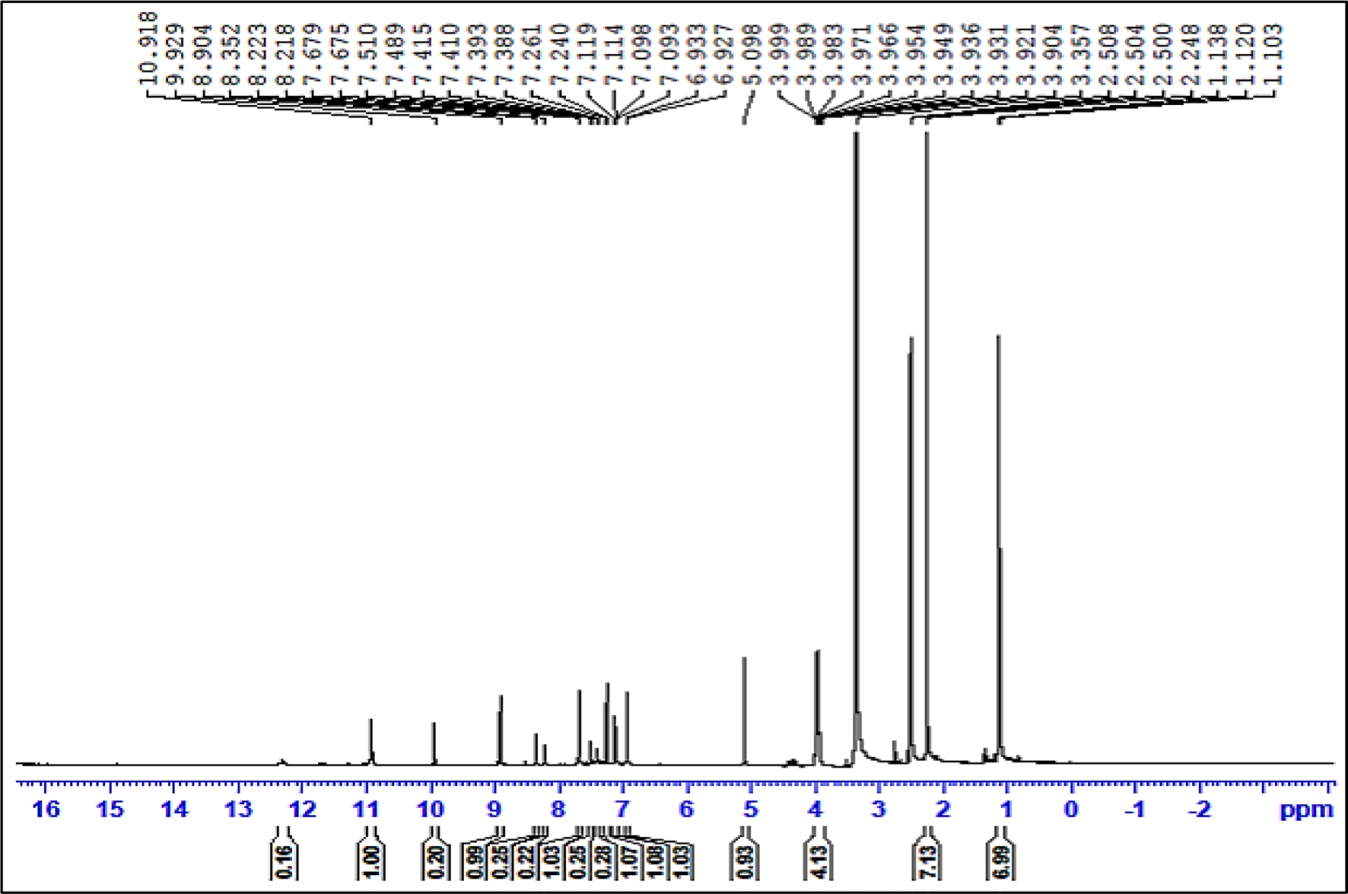
H1-NMR spectrum of DBIDDD

The proton nuclear magnetic resonance (^1^H-NMR) spectrum serves as a critical tool in discerning the molecular structure and confirming the successful synthesis of DBIDDD. The spectrum, illustrated in Fig. 2, reveals a plethora of characteristic peaks that shed light on the compound's intricate architecture.

#### NH protons

A distinctive peak appearing at 9.9 ppm is attributed to the NH proton of the indole ring, a signature resonance frequently observed for this moiety in a plethora of related compounds. Concomitantly, the pyridine ring's NH proton emerges at 8.9 ppm. The proximity of these peaks in the aromatic region accentuates the presence of nitrogen-bearing heterocyclic rings within the molecule [17]

#### Indole ring protons

The series of resonances in the range of 7.6 to 7.0 ppm distinctly demarcate the aromatic protons associated with the indole ring. Their multiplicity and chemical shifts align well with literature values for similar structures, thus reinforcing the incorporation of the indole fragment [18].

#### Methine proton

A notable singlet at 5.0 ppm corresponds to the methine proton, an isolated CH group within the molecule. This resonance is diagnostic of the CH's unique environment, distinct from the bulk aromatic and aliphatic regions.

*Ester Protons:* The ester moieties in DBIDDD manifest as two principal resonances. The CH2 protons adjacent to the oxygen atom of the ester group resonate at 3.9 ppm(J = 2 cps, J = 4.8 cps, J = 2 cps), indicating their shielded environment due to the electronegative oxygen atom. On the other end, the terminal CH3 protons of the ester chain exhibit a peak at 1.1 ppm (J = 7.2 cps, J = 6.8 cps), a typical chemical shift value for such methyl groups in an ester linkage [19].

#### Pyridine-bonded methyl protons

A significant peak at 2.5 ppm is ascribed to the methyl protons directly bonded to the pyridine ring. The chemical shift suggests their proximity to the electronegative nitrogen of the pyridine, causing a slight downfield shift compared to regular aliphatic methyl groups [20].

In summation, the 1H-NMR spectral data for DBIDDD resonates well with its proposed structure. Each peak offers a vital piece of evidence in the molecular puzzle, providing unequivocal support for the successful synthesis and purity of the compound.

### Mass spectral analysis of diethyl 4-(5-bromo-1H-indol-3-yl)-2,6-dimethyl-1,4-dihydropyridine-3,5-dicarboxylate (DBIDDD)

**Fig. 3:**
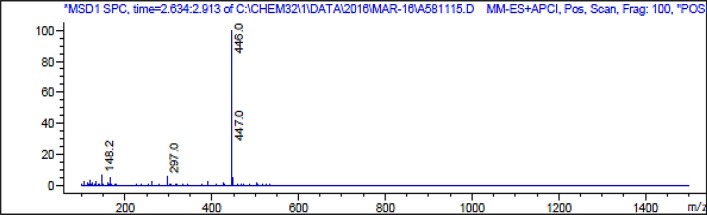
Mass spectrum of DBIDDD

The mass spectral analysis is an essential technique to not only confirms the molecular weight of a synthesized compound but also to infer potential fragmentation patterns that can provide invaluable structural insights. For DBIDDD, the detailed analysis of its mass spectrum, showcased in Fig. 3, provides intriguing results.

#### Molecular ion peak

The most definitive signature in the mass spectrum is the molecular ion peak, often denoted as M^+•^. For DBIDDD, this peak is distinctly observed at m/z 447. This value is in exact concordance with the calculated molecular weight of DBIDDD, implying the presence of the entire molecular framework without any fragmentation. This resonance confirms the precise molecular mass of the compound, underscoring the synthesis's accuracy and compound purity.

#### Base peak

Mass spectra often comprise a plethora of peaks corresponding to various fragment ions. Among them, the most intense peak is termed the 'base peak'. It serves as a key indicator of the most stable ion (or fragment) produced during the ionization process. For DBIDDD, the base peak is prominently registered at m/z 446. This peak, just one unit less than the molecular ion peak, could be attributed to the loss of a hydrogen atom from DBIDDD. Its high intensity signifies that this particular ion is highly stable and forms readily during the ionization phase.

In addition, while not explicitly mentioned in the initial description, one can expect other fragment ion peaks in the mass spectrum corresponding to various cleavages within the DBIDDD molecule. These peaks, when analyzed in tandem with other spectral data, can offer deeper insights into the molecule's structural nuances and possible fragmentation pathways.

In summation, the mass spectral data of DBIDDD, particularly the concordance of the molecular ion peak with its calculated molecular weight, serves as an unequivocal affirmation of the compound's successful synthesis and structural integrity.

### Preparation of inhibitor solution using diethyl 4-(5-bromo-1H-indol-3-yl)-2,6-dimethyl-1,4-dihydropyridine-3,5-dicarboxylate (DBIDDD):

#### Solution formulation

**Fig. 4:**
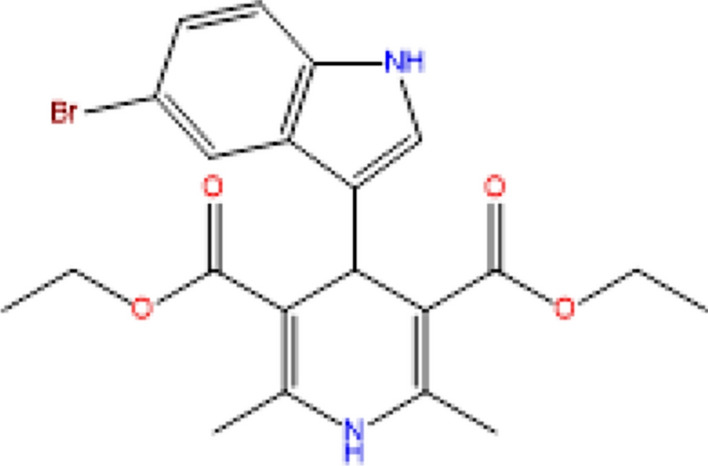
Structure of Diethyl 4-(5-bromo-1H-indol-3-yl)-2,6-dimethyl-1,4- dihydropyridine-3,5-dicarboxylate (DBIDDD)

- **Initial Dissolution:** The synthesized DBIDDD compound, renowned for its inhibitive properties, was selected as the primary component for the inhibitor solution. After a thorough verification of its structural and purity characteristics, a precise quantity of this compound was subjected to dissolution in 5 mL of concentrated sulfuric acid (Con.H2SO4). Using concentrated sulfuric acid ensures effective dissolution given its strong acidic and dehydrating nature, making it a solvent of choice for many compounds.
- **Volumetric Adjustment:** Post initial dissolution, the solution was carefully transferred to a 100 mL volumetric flask. This step ensures that the final solution volume is exact, thus guaranteeing the accuracy of subsequent dilutions. Deionized or distilled water was then added meticulously to the flask to achieve the 100 mL mark, ensuring a homogeneous solution. The use of a standard volumetric flask in this step underscores the commitment to maintaining precision throughout the process.
- **Preparation of Gradient Concentrations:** Leveraging the stock solution, a series of dilutions were then methodically executed to yield inhibitor solutions of varying concentrations. These ranged from 0.00004 M to 0.0002 M, providing a gradient that facilitates subsequent investigations, especially in assessing the inhibitor's efficacy across different concentration levels. Each dilution was carried out with attention to accuracy and precision, ensuring that the resultant solutions truly reflected the desired molarities.
- **Visualization:** For a holistic understanding of the entire process, Fig. 4 has been included to depict the chemical structure of DBIDDD. This visual aid not only acts as a reference point but also reinforces the relevance of the compound in the context of this work.

The inhibitor solution's preparation, anchored around the DBIDDD compound, was approached with a stringent focus on accuracy, precision, and reproducibility. Each step, right from the initial dissolution to the final dilutions, was executed with a meticulousness that underscores the scientific rigor of the process.

### Evaluation of inhibition efficiency via weight loss method

The weight loss method is a cornerstone analytical approach in corrosion studies, offering an empirical avenue to ascertain the rate of metal deterioration in the presence and absence of inhibitors. Given its directness and accuracy, this method remains indispensable in assessing the protective capabilities of novel corrosion inhibitors, such as the synthesized compound, DBIDDD.

1. **Coupon Preparation:** Mild steel coupons, standardized in their dimensions and weight, were subjected to a meticulous cleaning process. This ensures the removal of any surface impurities, oxides, or contaminants that could otherwise skew experimental results. After cleaning, the coupons were dried and accurately weighed, establishing a precise baseline weight for subsequent comparisons.
2. **Exposure to Corrosive Medium:** These prepared coupons were immersed in two distinct mediums:
3. Pure acidic medium, specifically H2SO4, serving as a control to gauge the inherent corrosion rate of mild steel.
4. Acidic solutions supplemented with varying concentrations of the synthesized inhibitor, DBIDDD, as detailed in Table 1. These solutions offer insights into the inhibitor's performance across different concentration gradients.
5. **Temperature Regulation:**To ensure consistency in the experimental conditions and replicate real-world scenarios, the immersion was carried out at a controlled temperature of 303 K. Such precision is pivotal, as temperature fluctuations can profoundly influence corrosion kinetics.
6. **Duration:** The immersion lasted for approximately 2 h. This duration was chosen based on literature precedents and offers a balance between achieving measurable weight loss while preserving the integrity of the coupons [21].
7. **Post-Immersion Analysis:** After the stipulated immersion time, each coupon was extracted, cleaned meticulously to remove any adhering corrosion products, dried, and re-weighed. The difference between the initial and final weights directly reflects the weight loss due to corrosion.
8. **Analytical Equations:** Employing the weight data before and after immersion, several critical parameters were computed using Eqs. (1–3):
9. **Erosion Rate (ER):** Quantifies the rate at which the metal undergoes corrosion.
10. **Surface Coverage (θ):** Represents the fraction of the metal surface protected by the inhibitor.
11. **Inhibition Efficiency (IE):** A metric that expresses the effectiveness of the inhibitor in preventing corrosion, typically given as a percentage.
  1 $${\text{Corrosion rate }}\left( {\rm P} \right) \, = \, \Delta {\text{W}}/\left( {{\text{D}} \times {\text{A}} \times {\text{T}}} \right)$$
  2 $${\text{I}}.{\text{E }}\left( \% \right) \, = \, \left( {{\text{W}}_{{{\text{blank}}}} - {\text{W}}_{{{\text{DBIDDD}}}} /{\text{W}}_{{{\text{blank}}}} } \right) \times {1}00$$

Where, W_blank_ = Loss of weight in H_2_SO_4_W_DBIDDD_ = Loss of weight in H_2_SO_4_ + DBIDDD

3 $${\text{Surface coverage }}(\Theta ) \, = \, \left( {{\text{I}}.{\text{E}}.} \right) \, /{1}00$$

The weight loss method, through its empirical rigor, offers an unequivocal perspective on the corrosion behaviour of metals and the protective prowess of inhibitors. In the context of DBIDDD, this method provides indispensable data on its efficacy as a corrosion inhibitor for mild steel in acidic environments.

### Electrochemical polarization and AC-impedance measurements

Electrochemical polarization measurements were studied for mild steel plates in H_2_SO_4_ and H_2_SO_4_ + DBIDDD to estimate the density of corrosion current, corrosion potential, and Tafel slopes b_cathode_ and b_anode_. [21].

4 $$\left( {\% {\text{ I}}.{\text{E}}.} \right) \, = \, \left( {\left( {{\text{I}}_{{{\text{corr}}}} } \right)_{{\text{plain acid}}} - \left( {{\text{I}}_{{{\text{corr}}}} } \right)_{{{\text{DBIDDD}}}} /\left( {{\text{I}}_{{{\text{corr}}}} } \right)_{{\text{plain acid}}} } \right) \, \times {1}00$$

$$\left( {{\text{I}}_{{{\text{corr}}}} } \right)_{{\text{plain acid}}} = {\text{ Density of corrosion current inH}}_{{2}} {\text{SO}}_{{4}}$$

$$\left( {{\text{I}}_{{{\text{corr}}}} } \right)_{{{\text{DBIDDD}}}} = {\text{ Density of corrosion current inH}}_{{2}} {\text{SO}}_{{4}} + {\text{DBIDDD}}$$

Impedance values were calculated at different frequencies. The data of charge transfer resistance, R_ct_ and the capacitance of double layer, C_d.1_, inhibition efficiency (I.E.) were measured via the following Eqs. (5 and 6) [24, 25].

5 $${\text{C}}_{{{\text{d}}.{\text{l}}}} = { 1}/{2}\pi {\text{R}}_{{{\text{ct}}}} {\text{f}}_{{{\text{max}}}}$$

6 $$\left( {\% {\text{ I}}.{\text{E}}} \right) \, = \, \left( {\left( {{\text{C}}_{{{\text{d}}.{\text{l}}}} } \right)_{{\text{plain acid}}} - \left( {{\text{C}}_{{{\text{d}}.{\text{l}}}} } \right)_{{{\text{DBIDDD}}}} /\left( {{\text{C}}_{{{\text{d}}.{\text{l}}}} } \right)_{{\text{plain acid}}} } \right) \, \times { 1}00$$

$$\left( {{\text{C}}_{{{\text{d}}.{\text{l}}}} } \right)_{{\text{plain acid}}} = {\text{ Capacitance of double layer inH}}_{{2}} {\text{SO}}_{{4}}$$

$$\left( {{\text{C}}_{{{\text{d}}.{\text{l}}}} } \right)_{{{\text{DBIDDD}}}} = {\text{ Capacitance of double layer inH}}_{{2}} {\text{SO}}_{{4}} + {\text{DBIDDD}}$$

The Three-electrode cell set up was employed for the investigation of both measurements.

### Surface analytical techniques for corrosion evaluation

Understanding the interaction between inhibitors and metal surfaces necessitates a suite of sophisticated analytical techniques that can delve into the nuances of these interactions. The combination of spectroscopic and topographical methods offers an intricate view of both the chemical and physical alterations on the metal surfaces post-treatment.

#### Spectroscopic snalyses

##### Fourier transform infrared (FT-IR) spectroscopy

FT-IR spectroscopy serves as an essential tool in discerning the chemical entities present on a surface. By probing the vibrational modes of molecular bonds, it provides a fingerprint of the functional groups and chemical moieties.

##### Application to DBIDDD

For this study, FT-IR spectra were obtained for the pure DBIDDD compound, establishing a reference spectrum. Furthermore, the spectrum of corroded mild steel immersed in a high concentration of DBIDDD was also recorded. By comparing these spectra, one can identify the potential chemical interactions or bond formations between the inhibitor and the steel surface.

#### Topographical investigations

##### Scanning electron microscopy (SEM)

SEM offers a magnified view of surfaces, enabling detailed visualization of morphology, texture, and potential alterations induced by external agents.

##### Instrumentation

The HITACHI MODEL S 3000 H SEM instrument was employed. Its advanced capabilities provide high-resolution imaging, ensuring that even minute details of the surface topography are captured.

##### Application

The surface morphology of the mild steel, post-treatment with DBIDDD, was meticulously analyzed. This provided insights into the extent and nature of corrosion, and the potential protective layer formed by the inhibitor.

##### Sessile drop method

Evaluating the wettability of surfaces is crucial, especially when understanding how inhibitors modify surface characteristics. The sessile drop method involves placing a liquid droplet on the surface and measuring its contact angle. This angle provides insights into the surface's hydrophilic or hydrophobic nature.

##### Application

For the mild steel plates treated with DBIDDD, the sessile drop method gauged how the inhibitor altered the steel's wettability. Such changes can have implications for corrosion resistance, as they influence how corrosive agents interact with the metal surface.

## Results and analysis

### Weight loss studies at various molarities-analysis of the anti-corrosive performance of DBIDDD in acidic medium

Utilizing the traditional gravimetric method, a time-honoured and effective means for understanding corrosion processes [22], the corrosive tendencies of mild steel in the presence of DBIDDD were assessed. The examination spanned multiple concentrations of DBIDDD, and the findings present compelling insights.

#### Inhibition efficiency (IE) trend

The data suggests a clear correlation between the concentration of DBIDDD and its effectiveness as a corrosion inhibitor. Specifically:

At lower concentrations of DBIDDD, a moderate inhibitory effect was observed. This is not unexpected; a lesser quantity of inhibitor molecules would mean fewer sites where adsorption can occur, leaving substantial portions of the metal surface vulnerable.

However, as the concentration of DBIDDD increases, there was a pronounced uptick in the inhibition efficiency. The peak value of this efficiency was noted to be 81.89% at a concentration of 2 × 10^-3 M at a temperature of 303 K. This is significant, demonstrating that a majority of the steel surface is protected under these conditions.

#### Corrosion rate analysis

A deeper insight into the corrosion rates further elucidates DBIDDD's anti-corrosive prowess:

At lower inhibitor concentrations, the corrosion rate of mild steel is relatively high at 0.9271. This corroborates the aforementioned point about a reduced protective effect at sub-optimal inhibitor concentrations.Impressively, at the apex concentration of 2 × 10^-3 M, the corrosion rate plummets to a mere 0.1679. Such a drastic reduction—beyond simply quantifying the inhibitor's efficacy—accentuates its potential commercial viability and relevance in real-world applications. The results are presented in the Table 1**.**

### Adsorption dynamics

The underlying mechanism responsible for this pronounced inhibitory effect is attributed to the adsorption of DBIDDD molecules onto the mild steel surface [23–25]. Several factors may be influencing this:

- Physical Adsorption: DBIDDD molecules may be adhering to the steel surface due to weak van der Waals forces.
- Chemical Adsorption: It's plausible that some form of chemical bond, albeit transient, is being formed between the DBIDDD molecules and the metal surface, facilitating enhanced protection.
- Molecular Orientation: The way DBIDDD molecules align themselves on the metal surface might be influencing the overall protective layer's compactness and robustness.

The characteristics of the Inhibitor Diethyl 4-(5-bromo-1H-indol-3-yl)-2,6-dimethyl-1,4- dihydropyridine-3,5-dicarboxylate are as presented in Table 2

**Table 2:** Characteristics of the Inhibitor

Name of the compound	Molecular Formula	Molecular weight, g	Melting Point	Colour of the Substance
Diethyl 4-(5-bromo-1H-indol-3-yl)-2,6-dimethyl-1,4- dihydropyridine-3,5-dicarboxylate	C_21_H_23_BrN_2_O_4_	447.32	160 C	Creamy white solid

### Adsorption isotherms

Adsorption isotherms are practiced to predict the relation between the surface coverage (Θ) and Molarity (C) of DBIDDD [21]. Few adsorption isotherms were evaluated to determine the adsorption mechanism [23–25]. The fit well was obtained with the Langmuir [26], Osagie [27], and El-Awady adsorption isotherms [28–30] by plotting the graphs between surface coverage against various concentrations of DBIDDD.

**Fig. 5:**
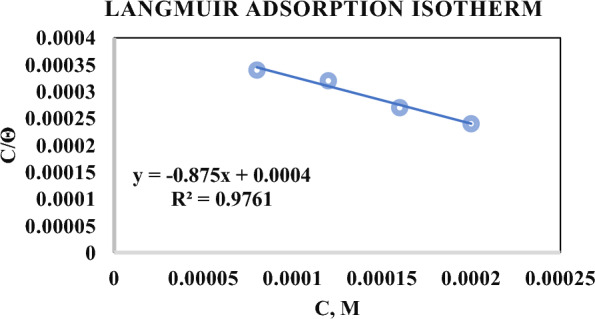
Langmuir isotherm Plot

The Langmuir adsorption isotherm [29] for **DBIDDD** of various molarities is plotted in Fig. 5. The values are given in Table 3. The regression value is nearer to 1 revealing the finest fitting [29]. The slope and intercept values are also indicated in the diagram.

**Table 3:** Data of Langmuir isotherm

[Inhibitor], M	Θ	C/Θ
0.00004	0.0709	0.00056
0.00008	0.2362	0.00034
0.00012	0.3700	0.00032
0.00016	0.5984	0.00027
0.00020	0.8189	0.00024

### Temkin’s adsorption isotherm

**Fig. 6:**
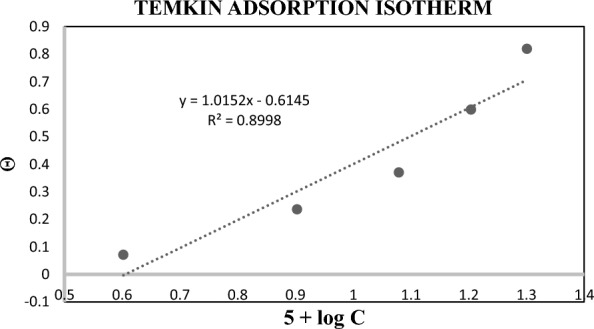
Temkin’s isotherm plot

Table 4 and Fig. 6 are given the concentration and molarity of the synthesized compound for plotting the Temkin adsorption isotherm. The R^2^ value predicts that Temkin adsorption is appropriate for synthesized organic compounds. The negative data of ‘a’ showcased the repulsion amongst the adsorption film of organic compounds [27].

**Table 4:** Data of Temkin’s isotherm

[Inhibitor], M	5 + log C	Θ
0.00004	0.6020	0.0709
0.00008	0.9030	0.2362
0.00012	1.0792	0.3700
0.00016	1.2041	0.5984
0.00020	1.3010	0.8189

#### *El-Awady *et al*., adsorption isotherm*

**Fig. 7:**
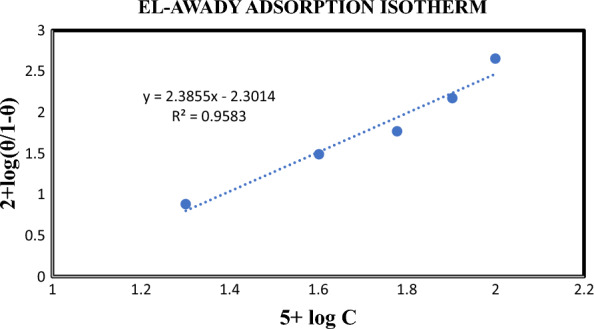
El-Awady*et al*., isotherm plot

Table 5 and Fig. 7 establish the values and linear plots of the El-Awady adsorption isotherm [28]. The graph given the regression factor value with 0.9583. It evidently predicts the inhibitor obeyed the isotherm.

**Table 5:** El-Awady*et al*., isotherm

[Inhibitor], M	5 + log C	θ	1-θ	(θ/1-θ)	2 + log(θ/1-θ)
0.00004	1.3010	0.0709	0.9291	0.0763	0.8825
0.00008	1.6020	0.2362	0.7638	0.3092	1.4902
0.00012	1.7781	0.37007	0.5875	0.5875	1.7690
0.00016	1.9030	0.5894	0.4016	1.4900	2.1731
0.0002	2.0000	0.8189	0.1811	4.5218	2.6553

#### Activation and Thermodynamic data for the inhibition process

Adsorption isotherm data gives the values of adsorption free energy, ΔG_ads,_ and equilibrium constant of the adsorption process K_ads_ [30] were calculated for various strengths of DBIDDD. The values are given in Table 6 [31–35]. The negative value of ΔGads showed the spontaneity of the adsorption progression. The value also obtained below the -40 kJ depicted the physisorption process between metal and inhibitor molecules [31].

**Table 6:** Data of thermodynamic adsorption

[Inhibitor], M	ΔG_ads_	K_ads_
0.00004	− 29.151	1907.76
0.00008	− 30.931	3865.50
0.00012	− 31.702	5249.20
0.00016	− 33.146	9312.70
0.00020	− 35.381	22,607.8

### Potentiodynamic polarization studies

**Fig. 8:**
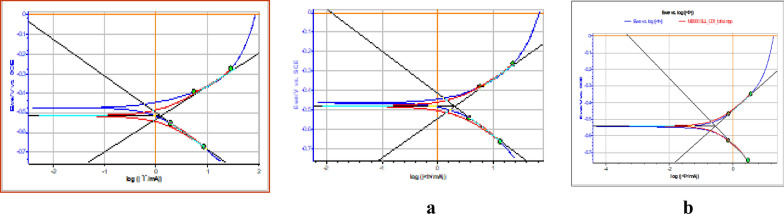
The curve of polarization of mild steel on acidic medium, **a** corroded mild steel at low concentration of DBIDDD, **b** at high concentration of DBIDDD

Potentiodynamic polarization studies data recorded on blank H_2_SO_4_ solution and different concentration solutions of DBIDDD were presented in Table 7**,** and plots are given in Fig. 8a, b. The result shows the decrease in the corrosion current values ((I_corrs_) from blank to the higher concentration of synthesized compound [32]. Appreciable changes are not noticed in the potential corrosion values (E_corrs_). Both the anodic slope (b_anode_) and cathodic slope values (b_cathode_) are altered. The inhibition potency reached to a maximum of 87.58%. This shows the combined mode of inhibition exhibited by the synthesized DBIDDD [33].

**Table 7:** Data of potentiodynamic polarization studies

[Molarity of Inhibitor] (M)	I_corrs.,_ mV	-E_corrs.,_ mV	b_anode,_ mV dec^−1^	b_cathode,_ mV dec^−1^	Efficiency Of Inhibition (%)
Plain Acid	2039.22	− 515.72	200.6	215.2	–
0.00004	1909.31	− 541.19	170.7	197.6	06.31
0.00020	253.204	− 480.57	159.4	186.2	87.58

### AC impedance experiment

**Fig. 9:**
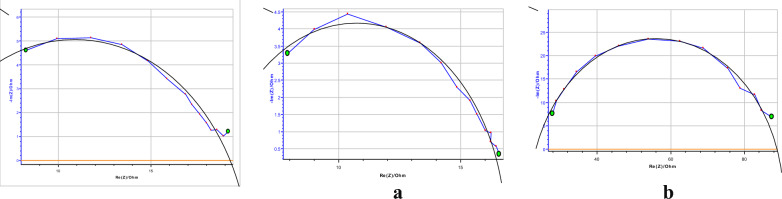
AC-Impedance diagrams of mild steel on acidic medium, **a**–**c** or roded mild steel at a low concentration of DBIDDD, **b** at a high concentration of DBIDDD

AC-impedance experiment is evident for the insoluble protective coating developed on the mild steel surface [34]. By this method, the charge transfer capacitance values R_c.t._ and double layer capacitance values were calculated and given in Table 8. The AC-impedance curves were pictorially denoted in (Fig. 9, b). The R_c.t._ values were raised with a rise in the molarity of DBIDDD and C_d.l._ values were decreased [34–36]. It founds the decrement of the dielectric constant and increment of the double layer thickness.

**Table 8:** AC-impedance experiment for mild steel in H_2_SO_4_ and DBIDDD in 1.0 M H_2_SO_4_

[Inhibitor], M	R_c.t._, Ohmcm^2^	C_d.l._, F/cm^2^	Inhibition efficiency (%)
Blank	11.57	0.155	––-
0.00004	16.70	0.141	09.03
0.00020	65.47	0.017	89.03

The maximum R_ct_ value of 65.47 Ω and minimum C_dl_ value of 0.017 F/cm^2^ are obtained at 0.0002 M concentration of DBIDDD with the maximum inhibition efficiency of 89.03% for the erosion of mild steel in 1.0 M H_2_SO_4_ shown in (Table 8).

### Surface analytical techniques

#### FT-IR spectral studies

**Fig. 10:**
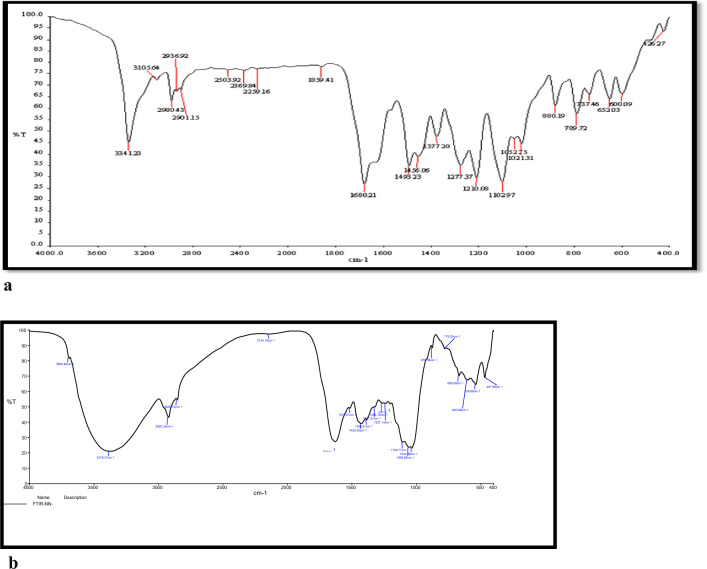
**a.**FT-IR spectrum of DBIDDD. **b** FT-IR spectrum of scratched mild steel at high concentration of DBIDDD

The Infra-Red bands of DBIDDD and the corroded sample in 0.0002 M of DBIDDD were shown in Fig. 10a, b, respectively. The FT-IR spectrum of the DBIDDD and the exterior layer on the mild steel plates after 2 h dipped in 1.0 M H_2_SO_4_ encompassing high concentration of DBIDDD are related in Table 9. The band at 3341 cm^−1^ is due to N–H stretching [37], and it is shifted to 3378 cm^−1^ and the aromatic C-H stretching vibration was lifted from 2980 to 2922 cm^−1^. The band that appears at 2901 cm^−1^ represents the existence of C-H stretching of ester group replaced in corrosion product to 2853 cm^−1^ [38–40]. The C = O group frequency changes from 1680 to 1615 cm^−1^. The 1493 and 1515 cm-1 bands are assigned to aromatic C = C stretching vibration [41, 42]. The bands at 1377 and 1264 replicate the shift in the frequency of C-H bending vibration [43–45]. The spectrum details act as supporting evidence for the formation of weak interaction between the Fe^2+^—organo inhibitor protective film.

**Table 9:** FT-IR spectrum of DBIDDD and scratched mild steel immersed in DBIDDD

DBIDDD	Scratched sample	Vibrational mode
3341	3378	N–H stretching
2980	2922	Aromatic C-H stretching
2901	2853	C-H stretching of ester group
1680	1615	C = O stretching
1493	1515	Aromatic C = C
1377	1264	C-H bending

### Morphological studies

#### Scanning electron microscopy (SEM)

**Fig. 11:**
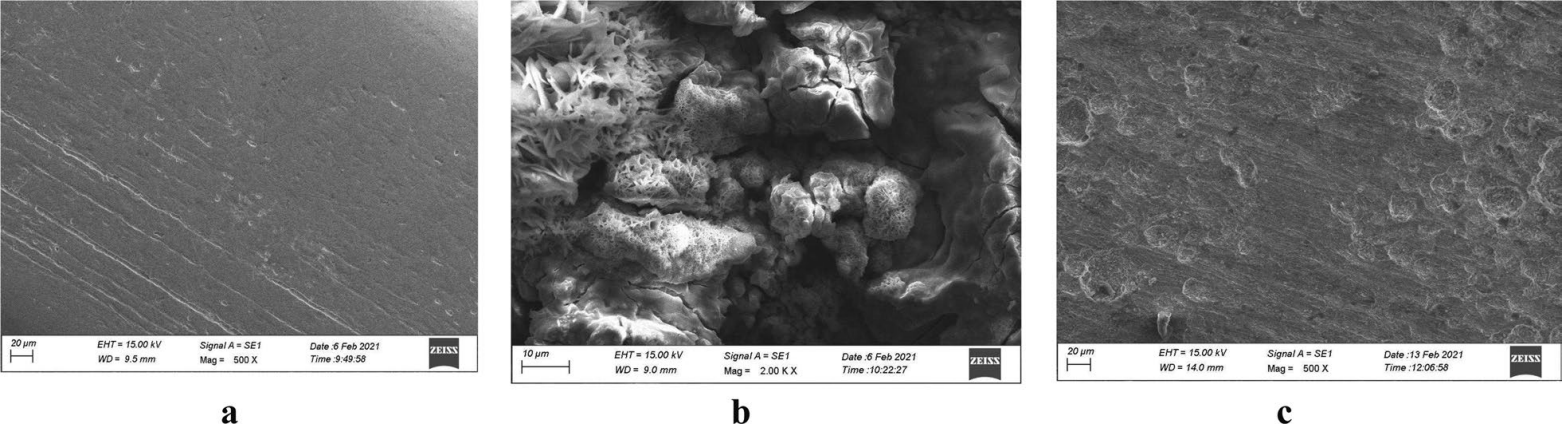
SEM micrograph of refined mild steel (**a**), mild steel dipped in acid (**b**), mild steel dipped in acid + 0.0002 M of DBIDDD (**c**)

The SEM pictures for refined steel, metal plate dipped in acid, and in the solution holding 0.0002 M of DBIDDD were exposed in Fig. 11a–c. The surface of mild steel is evener than refined steel (Fig. 11a). Associating Fig. 11b, c more depths and cracks were detected in Fig. 11b. The superficiality of the mild steel developed flatter in Fig. 11c compared with Fig. 11b. These imageries confirmed the formation of defensive film [46, 47] by the adherence of synthesized DBIDDD [47].

#### Atomic force microscopic experiment (AFM)

**Fig. 12:**
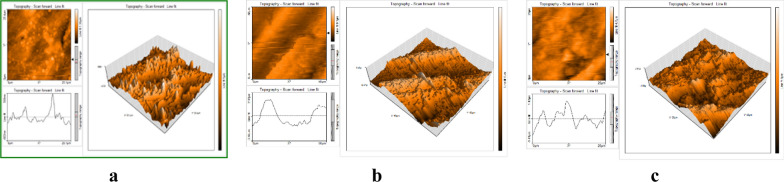
AFM (2D & 3D) pictures for refined mild steel (**a**), mild steel dipped in 1.0 M H2SO4 (**b**), and corroded mild steel dipped in 0.0002 M DBIDDD (**c**)

The 2D & 3D structural morphology of corroded steel is given in Fig. 12a. It indicates a porous structure with vast hollow voids. The outside of the mild steel plates covered with a self-protective coating is shown in Fig. 12b, c. The insoluble film formed a blockade and defended the plates from erosion [50]. The experiment data are given in Table 10 [48].

**Table 10:** Statistics of AFM

Specimens	Mean Roughness, nm	Root Mean Square Roughness, nm	Peak-Valley Height, nm
Refined Steel	112.89	141.56	1145.81
Strip in H_2_SO_4_	746.97	869.10	5915.98
H_2_SO_4_ + DBIDDD (0.0002 M)	341.06	428.73	3321.00

#### Contact angle measurements

**Fig. 13:**
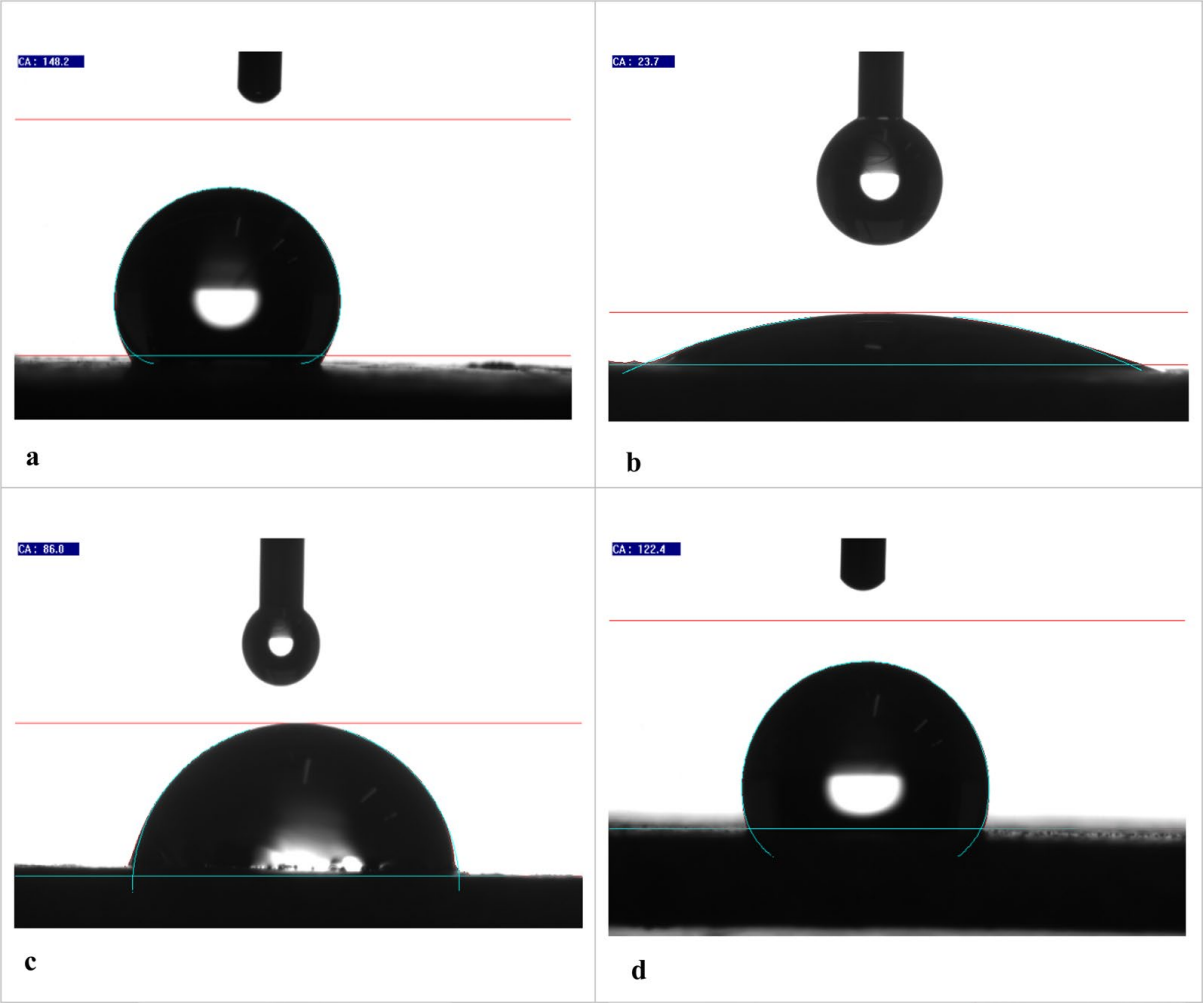
**a **Wetting angle for refined steel. **b** The wetting angle of corroded steel. **c** The wetting angle of corroded steel dipped in 0.00004 Mof DBIDDD. **d** The wetting angle of corroded steel dipped in 0.0002 Mof DBIDDD

The polished steel's aquaphobic nature or wettability characteristic, the mild steel coupons dipped in the plain acid solution. The plates dipped in the minimum and maximum concentrations of DBIDDD were assessed utilizing the contact angle experiment. The contact angle of more than 90° showed the aquaphobic nature [49]. The contact angle image of tested specimens is shown in Fig. 13a–d. The maximum value of 148.3^∘^ exhibited a high hydrophobic nature. Likening the contact angle data of uninhibited and inhibited solutions, the metal dipped in DBIDDD with the acid grasped the highest value, 122.4º. The metal strip dipped in H_2_SO_4_ exhibited a low contact angle value of 23.70^∘^. The contact angle images of solutions containing low and high inhibitor concentrations showed high contact angle values of 86.00∘ and 122.40∘, respectively [50] showing the coating of defended sheet by the DBIDDD through the lone pair of electrons present in the compound. This procedure involves blocking active sites on the exterior of mild steel like the aqua-lotus mechanism. The data were tabulated in Table 11.

**Table 11:** Statistics of contact angle experiment

System	[Inhibitor], M	Contact Angle, ^∘^
Refined Steel	–	148.3
H_2_SO_4_	–	23.70
H_2_SO_4_ + DBIDDD	0.00004	86.00
	0.0002	122.40

### Inhibition mechanism

The anti-corrosive performance of an organic inhibitor is due to the adsorption of the compound on the outward of the metal strip, which shields the metal exterior and thus reduces the deterioration process [51]. The adsorption of DBIDDD depends upon the structure of the compound. The synthesized compound has hetero atom, N, and aromatic π-electron ring systems. The electron-rich compound donates the electrons to the metal active sites and forms a barrier to protect against the metal's deterioration in acid environments [52].

### Antioxidant activity

**Fig. 14:**
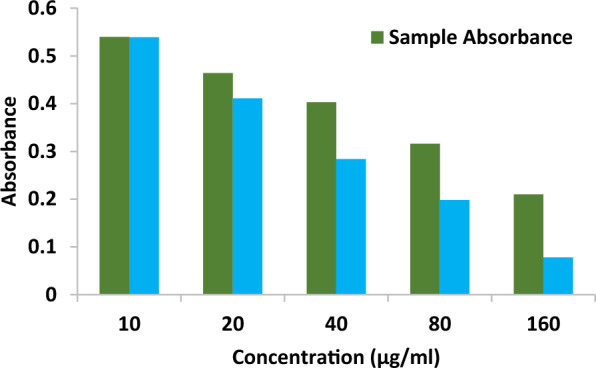
Concentration Vs Absorbance

**Fig. 15:**
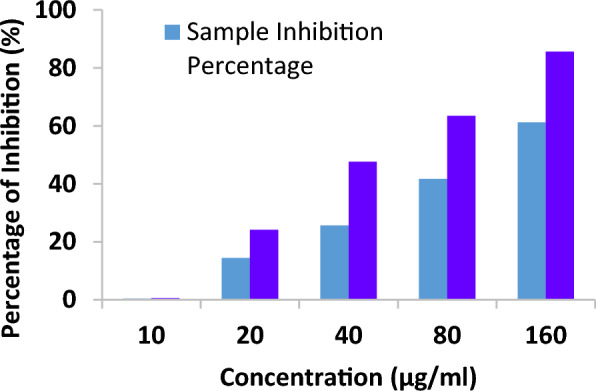
Concentration vs. Absorbance

**Fig. 16:**
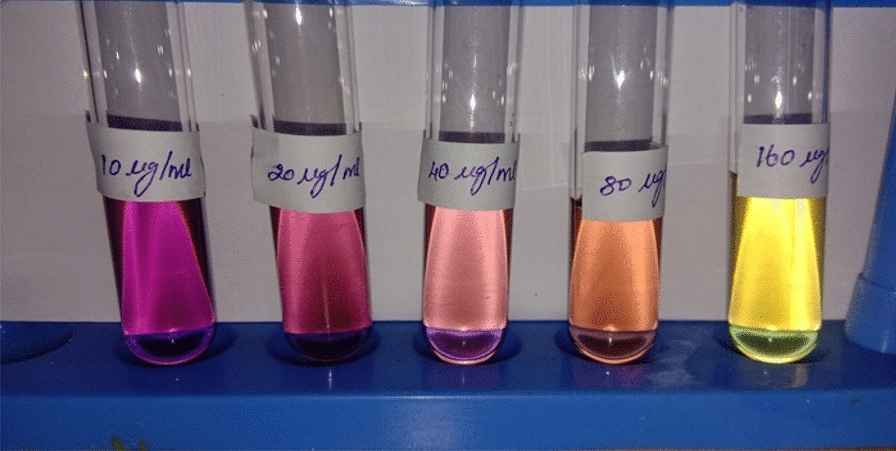
Antioxidant activity of ascorbic acid (Standard)

**Fig. 17:**
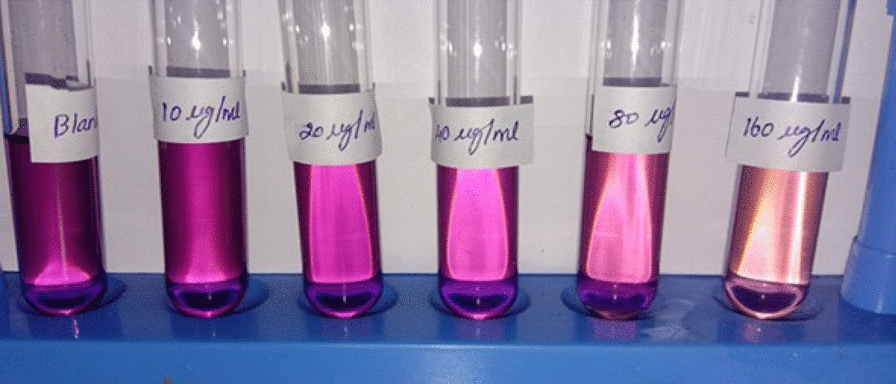
Antioxidant activity of ascorbic acid (Standard)

The compound's antioxidant activity (AA%) was investigated by DPPH free radical assay. A compound stock solution was made by liquifying 0.01 g in one mL of solvent (100 mg/mL); different strength was prepared, such as 10, 20, 40, 80 & 160 µg/mL. 1 mL of each sample solution was blended with 2 mL of DPPH, stored at a dark place, and permitted to react at room temperature for 30 min. The compound reduced the DPPH and changed its color from deep violet to light yellow. After half an hour, the absorbance was noted at 517 nm in UV–Visible spectrophotometry, and the proportion of radical scavenging activity, i.e., antioxidant activity, was computed. The control reading (Ascorbic acid) was analyzed by mixing 1 mL of solvent with 2 mL of DPPH reagent. The compound possessed an IC_50_ value of 113.964 ± 0.076, and the standard shows a value of 46.0377 ± 0.053. The antioxidant activity of the compound is comparatively higher than the positive standard. The results are represented in Table 12 and Figs. 14, 15, 16 and 17 [53–56].

**Table 12:** Antioxidant activity of the compound and standard

Sl. No	Concentration (µg/ml)	Standard Absorbance	Sample Absorbance	Standard % of DPPH Scavenged	Sample % of DPPH Scavenged
1	10	0.539	0.540	0.554 ± 0.002	0.369 ± 0.027
2	20	0.411	0.464	24.169 ± 0.004	14.391 ± 0.038
3	40	0.284	0.403	47.601 ± 0.003	25.645 ± 0.012
4	80	0.198	0.316	63.468 ± 0.001	41.697 ± 0.055
5	160	0.078	0.210	85.614 ± 0.003	61.254 ± 0.040
IC_50_ value				46.0377 ± 0.053 µg/ml	113.964 ± 0.076 µg/ml

### Evaluation of antimicrobial efficacy of the synthesized compound

To gauge the antimicrobial potential of the synthesized compound, a comprehensive screening was executed against an array of microbial strains. This evaluation embraced both Gram-positive, Gram-negative bacteria, and fungal pathogens, establishing a holistic perspective on its antimicrobial spectrum.

#### Gram-positive bacteria

*Staphylococcus aureus*: Often implicated in skin infections, respiratory infections, and food poisoning.

*Bacillus subtilis*: Commonly found in soil, it's a model organism in bacterial research.

#### Gram-negative bacteria

*Escherichia coli*: While some strains are benign, others can cause severe food poisoning.

*Pseudomonas*: Ubiquitous in the environment, certain species can be opportunistic pathogens, particularly in immunocompromised individuals.

#### Fungal pathogens

*Candida albicans*: A common fungus in the human flora but can cause infections in certain conditions.

*Aspergillusniger*: Commonly found in the environment, it can cause aspergillosis, especially in immunocompromised patients.

The agar dilution method was employed for this assessment, a tried-and-tested approach that allows for the determination of antimicrobial resistance or susceptibility by analyzing microbial growth in the presence of specific compound concentrations.Gentamicin, a potent aminoglycoside antibiotic, was utilized as a positive standard, serving as a benchmark for antimicrobial activity. The choice of Gentamicin underscores the seriousness of the assessment, as it's a widely recognized and potent antibiotic against a variety of bacterial infections.

The synthesized compound exhibited robust antimicrobial activity across the board, inhibiting the growth of all the tested pathogens.Impressively, its efficacy was not just on par with the positive standard (Gentamicin) but surpassed it. Such a finding underscores the compound's potential as a potent antimicrobial agent.

**Fig. 18:**
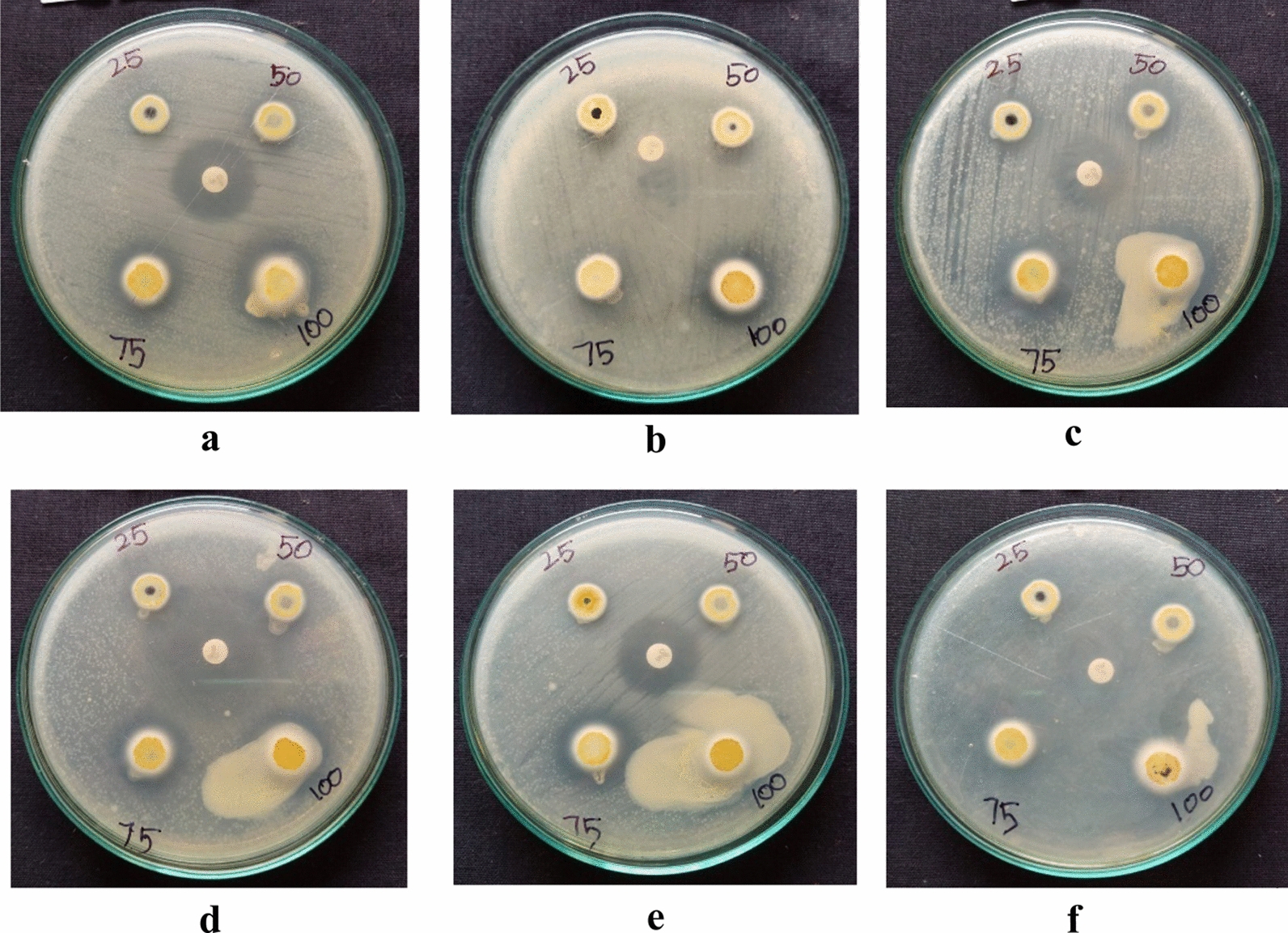
**a**–**f**: Agar plates show Zone of inhibition of DBIDDD against the bacteria and fungi pathogens: **a** *S. aureus* **b** *B. subtilis* **c** *E. coli* **d** *Pseudomonas* **e** *C. albicans* **f** *A. niger*

Detailed outcomes, including the zones of inhibition, which provide insights into the magnitude of the compound's antimicrobial prowess, are catalogued in Table 13 and visually represented in Fig. 18**.**

**Table 13:** Region of inhibition of the compound

Pathogens	Region of inhibition (mm/mL)				
	25 µL	50 µL	75 µL	100 µL	Control
*Staphylococcus aureus*	14	17	20	23	20
*Bacillus subtilis*	12	14	16	19	18
*Escherichia coli*	15	20	24	28	20
*Pseudomonas*	15	18	22	26	21
*Candida albicans*	15	20	25	30	22
*Aspergillusniger*	15	18	22	27	20

## Discussion

The genesis of our exploration was rooted in the systematic synthesis of Diethyl 4-(5-bromo-1H-indol-3-yl)-2,6-dimethyl-1,4-dihydropyridine-3,5-dicarboxylate (DBIDDD). Through rigorous procedural standards, the method was oriented towards achieving a high yield and molecular purity, a vital cornerstone for subsequent analyses. Advanced spectroscopic techniques, such as FT-IR, 1H-NMR, and mass spectrometry, were instrumental in delineating the compound's intricate molecular structure. These characterizations confirmed not just the molecule's integrity but also its purity, setting a foundational premise for the forthcoming phases of the investigation. DBIDDD showcased remarkable resilience in mitigating corrosion, especially in the aggressive milieu of acidic environments. It's noteworthy to emphasize the direct proportionality observed between the increasing concentration of DBIDDD and the enhancement in its corrosion-inhibitory attributes. This phenomenon, when observed within the lens of industrial applications, suggests that DBIDDD can play a pivotal role. Environments where mild steel, susceptible to corrosive agents, can significantly benefit from such protective mechanisms warrant further field-based assessments. Transitioning from a purely physicochemical realm to the biological sphere, DBIDDD’s interactions with microbial species were elucidated. Its broad-spectrum efficacy spanned across diverse microbial taxonomies, inclusive of both Gram-positive and Gram-negative bacterial strains. Its superior performance compared to Gentamicin, an established antimicrobial agent [57], adds further weight to its potential pharmaceutical applications [58–61]. The molecular interactions, whether through membrane disruption or inhibitory pathways, merit a deeper dive to fully harness its therapeutic potential. While the empirical data from this study is certainly illuminative, an in-depth mechanistic understanding is the linchpin to broadening its applications. How does DBIDDD interact at the molecular level, especially with metal surfaces? What are the fundamental pathways through which it hinders microbial growth? These are pivotal questions that the scientific community needs to address. Looking forward, a battery of tests, including in vivo toxicity studies, cellular interactions, and perhaps even proteomic analyses, will provide a more granular understanding. In summary, DBIDDD, with its multifaceted properties, emerges as a potential game-changer in both industrial and biomedical arenas. While laboratory-based findings provide a robust foundation, transitioning from bench to real-world scenarios is the eventual goal. Collaborative efforts, bridging the gaps between molecular chemists, industrial engineers, and biomedical scientists, will be the key to unlocking its full potential, ushering in novel applications that were hitherto uncharted.

## Conclusion & future directions

In our comprehensive study of diethyl 4-(5-bromo-1H-indol-3-yl)-2,6-dimethyl-1,4-dihydropyridine-3,5-dicarboxylate, the compound has unequivocally demonstrated its multifaceted applications and potential in various sectors. Utilizing state-of-the-art spectral methodologies, such as FTIR, NMR, and Mass spectrometry, we validated the compound's structural integrity, consistency, and purity.Its potent capability as a corrosion inhibitor, particularly on mild steel in aggressive acidic environments, herald’s significant implications for sectors reliant on metallic longevity. Empirical evaluations reflect a direct correlation between compound concentration and corrosion mitigation. A zenith inhibition efficiency of 81.89% at 2 × 10^–3^ M concentration underlines the compound's industrial relevance.In the biomedicinal context, the compound showcased exemplary antimicrobial attributes against a spectrum of bacterial strains and fungal pathogens. The fact that it surpassed Gentamicin, a well-regarded antibiotic, in efficacy denotes its potential in antimicrobial therapeutics. Its antioxidant prowess, as evidenced by the DPPH assay, further affirms its potential therapeutic utility.

The synthesized compound's multifunctionality warrants a deeper, more nuanced exploration. A strategic trajectory would involve molecular-level mechanistic studies to elucidate interaction dynamics. This would not only enhance our understanding but also refine application-specific modifications.Given the superior antimicrobial metrics observed, there's a compelling case for progressing into pharmacokinetic and pharmacodynamic analyses. This could potentially position diethyl 4-(5-bromo-1H-indol-3-yl)-2,6-dimethyl-1,4-dihydropyridine-3,5-dicarboxylate as a potent weapon against the burgeoning global challenge of antimicrobial resistance. Its antioxidative capacity further augments its profile for prospective therapeutic or even nutraceutical applications.On the industrial front, extensive trials under varied environmental conditions would ascertain its versatility as a corrosion inhibitor. An interdisciplinary approach, with synergetic collaborations amongst chemists, biotechnologists, and materials scientists, will be pivotal in harnessing the compound's full application spectrum, ensuring a transition from benchtop research to real-world implementation.

## Data Availability

All data generated or analysed during this study are included in this published article and its supplementary information files.
